# Comparison of quality control for trauma management between Western and Eastern European trauma center

**DOI:** 10.1186/1749-7922-3-32

**Published:** 2008-11-19

**Authors:** Stefano Massimiliano Calderale, Raluca Sandru, Gregorio Tugnoli, Salomone Di Saverio, Mircea Beuran, Sergio Ribaldi, Massimo Coletti, Giorgio Gambale, Sorin Paun, Livio Russo, Franco Baldoni

**Affiliations:** 1 Emergency and Trauma Surgery Unite, Maggiore Hospital Trauma Center, Bologna, Italy; 2 Gruppo Aperto per lo Studio del Trauma (GAST), Dipartimento di Emergenza e Urgenza, Policlinico Umberto I, Sapienza – Università di Roma, Italy; 3 Departamentul de Chirurgie Generala, Spitalul de Urgenta Floreasca, Bucharest, Romania

## Abstract

**Background:**

Quality control of trauma care is essential to define the effectiveness of trauma center and trauma system. To identify the troublesome issues of the system is the first step for validation of the focused customized solutions. This is a comparative study of two level I trauma centers in Italy and Romania and it has been designed to give an overview of the entire trauma care program adopted in these two countries. This study was aimed to use the results as the basis for recommending and planning changes in the two trauma systems for a better trauma care.

**Methods:**

We retrospectively reviewed a total of 182 major trauma patients treated in the two hospitals included in the study, between January and June 2002. Every case was analyzed according to the recommended minimal audit filters for trauma quality assurance by The American College of Surgeons Committee on Trauma (ACSCOT).

**Results:**

Satisfactory yields have been reached in both centers for the management of head and abdominal trauma, airway management, Emergency Department length of stay and early diagnosis and treatment. The main significant differences between the two centers were in the patients' transfers, the leadership of trauma team and the patients' outcome. The main concerns have been in the surgical treatment of fractures, the outcome and the lacking of documentation.

**Conclusion:**

The analyzed hospitals are classified as Level I trauma center and are within the group of the highest quality level centers in their own countries. Nevertheless, both of them experience major lacks and for few audit filters do not reach the mmum standard requirements of ACS Audit Filters. The differences between the western and the eastern European center were slight. The parameters not reaching the minimum requirements are probably occurring even more often in suburban settings.

## Background

International research on trauma is increasingly focusing on the quality improvement in the management of this emergent public health problem. The first priority for quality improving is the inter- and the intra-hospital quality control. After identifying the troublesome issues through the inter-facility control, a detailed verification of intra-facility clinical problems can give valid solutions to improve the quality of care. The quality level of a service is defined, according to Donabedian's definition, by three essential aspects: the structure, the process and the outcome. [1,2] In our study, the structures analyzed in comparison – an Italian and a Romanian Level I trauma center and their resources, are very similar.

Maggiore hospital is the referral Trauma Center in the Bologna province and Emilia-Romagna region, since 1980's. Its catchment area encompasses approximately 1,500,000 people in Emilia-Romagna. The Urgenta hospital has been an emergency and trauma center since 75 years, with availability of special structures and a team for the management of trauma patients. Its catchment area includes 1,500,000 people, inhabitants of Bucharest but also of the provinces of the South and South-East of the country.

The extent of the two hospitals are similar (Table 1). The Emergency Departments of the two centers are organized in a similar way, and they both meet the criteria of the American College of Surgeons for the level I trauma centers. The differences are in the organization of operating rooms – exclusively dedicated for trauma and emergency cases in the Romanian hospital and available on a 24/7 basis, while in the Italian hospital the OR is shared with Vascular and Urologic surgery for urgent cases – and the presence in the ED of the surgeon (present in the ED on a 24/7 basis in Bucharest, on call from the surgical ward for the ED in the Bologna hospital).

**Table 1:** Hospitals' description

Criteria	Bologna	Bucharest
Number doctors	400	476

Number nurses	1.100	780

Beds	600	700 (available up to 1000)

Trauma surgery	General and emergency surgery Emergency surgery: 5 surgeons, 12 beds	3 general surgery units: 33 surgeons, approx. 180 beds

ICU	10 beds	SICU: 14 beds Major trauma: 8 beds

Ambulance services	Centralized: 118	Centralized, public: 961 Private: varied

Emergency department	3 boxes: emergency physicians 1 emergency room 3 rooms for short observation Radiology Laboratory TC Angiography on call	1 emergency room: emergency physicians, general surgeon 1 box: medical emergencies 1 box: orthopaedics Radiology Laboratory TC Angiography on call

Presence of the trauma surgeon	24/24 hours on-duty	24/24 hours in the ED

Trauma team leader	Anesthesiologist	General surgeon

Doctor who admits the patients	Minor trauma: emergency physician Major trauma: anesthesiologist	Minor trauma: emergency physician Major trauma: general surgeon

SICU (Surgical Intensive Care Unit)	No	Yes (12 beds/general surgery unit, 1 nurse/each 2 beds, anesthesiologist 24/24 hours)

For the evaluation of the process and the outcomes we evaluated the average aims and resources according to the 22 recommended minimal audit filters for trauma quality assurance by The American College of Surgeons Committee on Trauma (ACSCOT) as reported in the Maryland Trauma Registry, Education and Prevention Committee, Maryland Trauma Registry Data Dictionary [3].

The aim of this study was to obtain results to be used as the basis for recommending and planning changes in the two trauma systems for a better trauma care in the two countries.

## Materials and methods

We evaluated and analyzed all the major trauma patients admitted between January 1st 2002 and June 30th 2002 in two Level I trauma centers.

The two hospitals included in the study are the Maggiore Hospital in Bologna, Italy and the Spital de Urgenta Floreasca in Bucharest, Romania. Both these institutions are classified as Level One trauma center, according to the criteria of the American College of Surgeons. (Table 2)

**Table 2:** Comparison of the criteria for trauma centers level I Ospedale Maggiore-Bologna versus Spital Urgenta-Bucharest

Criteria	Bologna	Bucharest
Catchment area	1,500,000 people	1,500,000 people

Nr. trauma per year	Aprox.20,000 visited	Aprox.12,000 admitted

Major trauma per year	221	201

Neurosurgery/24 hours	On-call	On-duty

Heart surgery/24 hours	On-cal; no heart surgery unit	On-duty

Burn unit	Under construction	Under construction

Neonatology	No	No

Operating theatre for trauma	Yes (available at request)	Yes (six, for trauma)

During the year 2002, approximately 20,000 trauma patients have been admitted to the Emergency Departement of Maggiore hospital. In the same period, the trauma patients admitted to the Urgenta hospital were 12,000. Major trauma patients (ISS >16) were 221 in Bologna and 201 in Bucharest.

Neither the Bucharest hospital, nor the Bologna hospital have a complete data-base for all their units. That is why we used the data base from the Trauma ICU of the Italian center and the archive of the hospital of Bucharest. In our study, all the trauma patients with ISS>16 admitted in the ED of the Maggiore hospital and transferred to the ICU were included; 11 patients admitted to other units than ICU were excluded.

At the Urgenta hospital of Bucharest, we analyzed all the trauma patients with an ISS>16, admitted to any department. The database of the Emergency Department of this hospital does not include the ISS. It was calculated retrospectively, using the data from every single clinical record. The Bologna series included 92 trauma patients, with ISS >16, from a total of 113 patients admitted to ICU in the period of the study of six months. There were 245 admissions to the ICU in the whole year 2002, out of them 221 had an ISS >16. In the Romanian center, the trauma patients with ISS>16 admitted in the six months period were 107, out of a total of 193 admissions. We excluded 17 patients because of the lacking of data, and we analyzed 90 trauma patients with ISS>16. The total number of major trauma admitted at this center in the year 2002 was 426 (201 with ISS >16).

Every phase of the management of these patients was analyzed according to the ACS 22 audit filters. [3]

## Results

The 90 trauma patients of the Romanian group had a mean ISS of 29.2 and the 92 patients of Bologna a mean ISS of 28.7. In both groups, there is a strong prevalence of male over female patients: 21/90 (23.3%) were female and 69/90 (76.7%) were male patients in Bucharest and 15/92 (16.3%) women and 77/92 (83.7%) men in Bologna. The mean age was 44 years in the group of Bucharest and 42.5 years in the Italian patients.

The mechanism of trauma was mostly represented by traffic crashes: 72.2% in the Romanian and 76.1% in the Italian series.

In our study we were not able to analyze the filters A1 and A2 (regarding the prehospital phase) because of the inability to obtain the needed information. Neither the time of ambulance's arrival, nor the presence of the medical report in the prehospital phase were reported in the databases of the two hospitals.

We excluded as well the filter A6 (surgical treatment of abdominal gunshot wounds) because in none of the two groups there were patients with GSW trauma.

We also could not evaluate the A14 filter (reintubation after less then 48 hours from extubation) for the hospital of Bologna, because missing data about extubation and reintubation.

According to the audit of the ACS, all the trauma patients with a GCS<8 should be transferred from the ED already intubated (A5). The patients with a GCS<8 were 31/92 (33.7%) at Bologna and 37/90 (41.1%) at Bucharest. All these patients were intubated at exit from the ED.

The filter A3 is concerning the head trauma management (head CT scan done within the first 2 hours in ED for patients with head trauma and GCS<14). The patients with head trauma and GCS<14 not dead within the first 2 hours from the arrival, were 54 at Bologna and 47 at Bucharest. After excluding the patients where the time of the CT was not registered, this filter resulted to be properly applied in 90.2% of the cases in Bologna and 93.6% in Bucharest.

The filter A8 suggests that all the patients with a subdural or epidural haematoma should undergo craniotomy within the first 4 hours from the arrival in the ED. The trauma patients who received a craniotomy were 14/92 (15.2%) at Bologna and 19/90 (21.1%) at Bucharest. The craniotomy in the first 4 hours was done in 7/14 (50%) of the patients from Bologna and 12/19 (63.2%) at Bucharest.

The surgical management of the abdominal trauma is verified by the filters A7a and A7b. The abdominal trauma with hypotension – 19/92 (20.7%) of the Italian patients and 21/90 (23.3%) of the Romanian – was treated surgically within the first hour in 100% of the cases in Bologna and in 76.2% of the cases in Bucharest Hospital. The data about the time of the operation were completely recorded in both of the groups. Surgical therapy was not postponed later than 4 hours from the patient's arrival in the ED in both hospitals, for most of the patients requiring laparotomy. The patients who needed a laparotomy were 28/91 (30.7%) in Bologna (one patient had missed data) and 55/90 (61.1%) in Bucharest. Early laparotomy (within 4 hours) was performed in 89.3% of the cases at Bologna and in 100% at Bucharest.

About the length of stay in ED (A9b), none of the patients of both of the groups exceeded the limit of 6 hours suggested by the ACS.

The filter A17a verifies the non planned surgical procedures. Only 10/53 (18.9%) of the operated patients of Bologna and 12/58 (20.7%) in Bucharest required a non planned surgical procedure. The unexpected admission to the ICU are in the filter A17b. Out of the 92 patients admitted to the ICU in the Italian hospital, only 5 (5.4%) required an urgent admission. In the Romanian hospital, the patients admitted to the ICU were 69, out of them 3 (4.4%) were admitted unexpectedly.

The filter A11 indicates the need of early definitive surgical care for trauma patients. It establishes that thoracic, abdominal, vascular and cranial surgery have to be performed within the first 24 hours from the arrival to the hospital. The patients who underwent this kind of operations were 38/92 (41.3%) in Bologna and 48/90 (53.3%) in Bucharest. A number of 28 (73.7%) patients were operated within the first 24 hours in Bologna and 34 (70.8%) in Bucharest.

About the transfers from another hospital (transfers in, A9a), there were 7/92 (7.6%) transfers to the hospital of Bologna and 67/90 (74.4%) to Bucharest. The severe lack of data about transfers, occurring in 50.7% of the cases of Bucharest and 100% of Bologna, precluded any further comparative analysis. In the Romanian group, 36.4% of the transferred in cases had a delayed time higher than 6 hours. The limit of 6 hours is suggested by the ACS.

The transfers to another hospital (transfers out, A9c) occurred in 0% of the cases in Bologna and 1% in Bucharest (only one paediatric patient, transferred after resuscitation, before the 6 hours limit, into paediatric hospital).

According to ACS' audits, trauma patients should always be admitted to the hospital by a surgeon (A12). The trauma team leader in the Romanian hospital is the general surgeon with competence in trauma, while in the Italian hospital, this role is referred to an anesthesiologist. However, even if the minor trauma patients are admitted in both of the hospitals by the emergency physician, major trauma is always managed by trained team with specific expertise in trauma.

We reported a not statistically significant difference between the rate of complications (A15) in the two groups: 69.6% in Bologna and 53.7% in Bucharest.

The analysis of the mortality (A16), has been limited to only the ICU mortality of the Italian hospital, reported as high as 21.7%, while in the Romanian group the reported overall in-hospital mortality was 47.8%.

The patients not operated or admitted to the ICU within the first hour from their arrival in the hospital and not dead during this time were 89/92 (96.7%) in Bologna and 45/90 (50%) in Bucharest. Only in 15.7% of the Italian and in 20% of the Romanian patients an hourly chart documentation has been found (A4.2)

The audit filter A10 verifies the early treatment (within 8 hours after ED arrival) of open fractures of the long bones due to blunt trauma. The filter was fulfilled in 6/13 (46.2%) patients with open fractures of Bologna and 13/21 (61.9%) patients of Bucharest.

About the fixation of femoral diaphyseal fractures in the Emergency Department, A13, the fulfillment rate is even lower: 20% (2/10) in Bologna and 40% (2/5) in Bucharest.

## Discussion

The filters analyzing the preclinical phase, the treatment of gunshot wounds and the early reintubation of trauma patients were excluded from the study. The low incidence of penetrating versus blunt trauma is consistent with the trauma epidemiology in European countries. Unfortunately no complete information about extubation/reintubation were available, although this paramenter is strongly associated with the outcome of the trauma patient. [4-6]

Diagnosis and early surgical therapy of head trauma are performed in both the centers, reaching the international minimum standards required. [1,6-9]

The same results have been reached in the management of abdominal trauma. The time to laparotomy is one of the strongest predictive factors affecting the overall outcome of the patients with abdominal trauma, and it contributes to avoid the preventable deaths. [10-14] The low proportion of patients fulfilling the standard for this filter affected the significance of the differences between the two groups.

Satisfactory results have been obtained as well for the airway management in unconscious patients. Intubation in patients with brain injury is essential; it offers significant advantages, especially if done early, and is often a lifesaving measure. [15-17] The optimal results obtained (100% in both the groups from the Italian and Romanian hospital) can be explained by the steady presence of the anesthesiologist in the ED, being leading part of the trauma team. This is the reason because all the patients with GCS = 8 were always already intubated in ED, before to be transferred, (if not already intubated in the preclinical phase). Similar studies show also good results for this filter [6]

The minimum time period of ED stay both in Romanian and Italian hospital, showed the efficiency of organization of the clinical services and departments, effective interdisciplinary teams cooperation and communication, effective utilisation of the resources. The significantly reasonable results reached in the transfers-out confirm the availability of effective resources.

The surgical decision-making in the Bucharest center is more oriented towards the early definitive surgical treatment of abdominal, thoracic and head injuries, while in Bologna Damage Control Surgery is preferred in selected unstable patients [18]. However the experiences from the literature, usually reported higher rate for application of this audit. [19]

The limited accessibility of CT scan (shared with other services) in the Romanian Hospital, could partially explain the reason for doubling of need for laparotomy when mechanism of injury and apparent severity of illness is the same in the two populations.

The differences between the two groups involve as well the transfers-in. This result should suggest a prompt improvement of the trauma systems in the two countries. The severe lack of information about transfers-in from both the hospitals is an evidence that the trauma systems are not yet homogeneous, and demonstrates an insufficient attention towards this time period of patients' management and a low efficacy of interhospital collaboration. A large amount (63.6%) of the transfers-in the Romanian group was not done according to the audits of the ACS, but later than the first 6 hours from the arrival in the first hospital. Considering that 75% of the Romanian patients were referred from other hospitals, it is very significant. We have to underline, that the patients transferred to the hospital of Bucharest came not only from a well defined region around Bucharest, but also from more than 300 km far away regions. These findings could eventually suggest that the problems connected to the transferring risks – increasing with the time – are not yet well considered. [20-22]

The trauma leader is the anesthesiologist in Bologna, while in Bucharest, this role is committed to general surgeons with special expertise in trauma. Thus, the Romanian hospital is closer to the American model, while the Italian system is similar to the European countries organization. [8,11,20,23-25] Since there is not yet an homogeneous opinion or evidences in which one of these models is preferable, no definitive conclusions can be made.

During our research we noted some lacks in the two centers. One problem concerns the specific complications. It is well known that major complications during hospitalization can cause an over-utilisation of resources, an higher mortality, a longer hospitalization period and increased costs. [26,27] Both in Bologna and in Bucharest, more than half of the patients developed complications, with higher frequency than other reported results from the literature. However, the patients included in our analysis has higher trauma severity score than the other groups reported from the literature. [7,26,28-30]. The higher complication rate in the Italian group (70% vs 54%) could be explained since those patients most likely to have a complication in Romanian group, they prior to developing a complication

However, the lacks in recording the complications in the Romanian group, affected the validity of application of this filter. Nevertheless, the low rate of unexpected surgical operations or admissions to the ICU, A17a and A17b, is an indirect evidence that, to date, the more severe complications are rarely occurring. The low number of unexpected admissions to the ICU, gives also an estimation of the level of care, of the human resources quality, of the satisfactory interdisciplinary collaboration within the hospital.

Mortality itself is a controversial measure in judgement of the quality of trauma center quality and efficiency [31]. However It is one of the ACSCOT filters. The results of our research in the analysis of mortality rates have to be discussion. The overall mortality rate of the Maggiore hospital of Bologna is lower than the mortality reported by most of the studies on major trauma mortality. [7,32,33] (Table 3). Yet, this percentage includes only the deaths occurring during the ICU stay. We could not find any information about death rate of trauma patients admitted in the other departments of the Italian hospital – probably low, nor about late mortality of the ICU patients after their transfer to step-down ward. The mortality rate reported in the Urgenta hospital of Bucharest is the overall mortality, during the whole period of hospitalization. It includes, therefore, also the late mortality.

**Table 3:** Percentage of trauma deaths according to ISS severity groups:

Severity groups (ISS)	Mortality Bologna	Mortality Bucarest	Mortality international literature
Mean value	21,7%	47,8%	26,6% – 41,3%

15–24	7,1% (2/28)	16,6% (5/30)	16,8%

25–49	26,6% (16/60)	62,5% (35/56)	46,4%

50–75	50% (2/4)	75% (3/4)	75,8%

Many international researchers found that mortality rate is amenable to ranges of ISS values [33] more than to single unique ISS values. [15,29,34-36] More often, other predictive factors or their combination are considered. We found from the literature the following significant predictive factors: ISS (>16, >18, >25), associated head trauma [2,37,38], age (especially >60 years) [33], transfers [32,39] improper transport from the place of the crash (taxi, private car of a witness, police car, not equipped ambulance, etc.) [40]

As regards our data, it comes out that the ISS, the head trauma and the age are slightly higher in the Romanian group than in the Italian (mean ISS value of 29.27 vs 28.71, mean age of 43.91 vs 42.46 years, mean GCS of 9.49 vs 10.32, with a percentage of 41.1% of patients with GCS<8 in Bucharest and 33.7% in Bologna).

The high number of head trauma patients in the Romanian group could indicate a more severe injury. However, if transfer is delayed and/or pre-hospital care is inadequate in Romania, patients with similar injuries would appear to have a greater head injury than those in Italy because of the difference in pre-hospital care, since it contributes to the ultimate GCS on presentation to the emergency department.

The patients over 60 years – where the death rate is highest [26,33,37] – are equally occurring in both groups, and it clearly shows that the age has no influence on the difference between the death rates. The transfers to the Romanian hospital were 10 times higher than those received by the Italian center, but the mortality of the Romanian patients admitted directly in the trauma center is almost the same of the patients transferred from other hospitals (47.82% against 47.76%). The factor strongly influencing the mortality seems to be the lack of an equipped ambulance and of a rescue team in 62.5% of the Romanian patients. Furthermore, according to other reports, also the combination of injury mechanism and the ISS has a high predictive value [15,33], since injured pedestrians have the lowest survival rate. In the Romanian group there were 22/90 (24.4%) pedestrians involved in traffic crashes, while in the Italian group these kind of patients were 6/92 (6.5%). We can therefore assume that the lack of registration of late deaths in Bologna, the major incidence of head injury and pedestrian victims of traffic crashes in the Romanian group, the inadequate management in pre-hospital phase of the Romanian group could explain this difference of mortality percentages between the two centers.

Another problem has been the hourly chart documentation; this procedure can improve the trauma service [41]. For both hospitals, this audit rarely occurs. This could be due to the absence, in both centers, of a recording nurse, as proposed by some authors. [20,23]

Finally a further major problem is the treatment of fractures. Neither early surgery of open fractures of the long bones, nor the femoral fracture fixation are not usually performed in the two centers, although slightly better results were achieved in Bologna. None of the two centers met the current international standards. [13,42-48]

Mortality in the Romanian hospital is twice that of the Italian hospital, despite similar type of patients, similar access to care, and similar approaches the injured patient. This cohort study demonstrates that unless one is able to control for the delivery of care throughout, from injury to recovery, that analysis of one component, in this case the hospital care of the patient, can be significantly distorted by other uncontrolled components of the care.

## Conclusion

The hospitals included in our study are both level I trauma centers. Each is, in their own country, a reference model for all the suburban hospitals that manage trauma patients.

The Bologna and Emilia Romagna region setting is organized according to the Hub and Spoke model.

We distinguished and classified the results in four groups according to the filter application percentages (very low, low, good, optimal) (Table 4)

**Table 4:** Results grouped according to the application percentages.

Application percentage	Nr. Filters Bologna	Nr. Filters Bucharest
0–25%	3	1

25–50%	2	2

50–75%	2	5

75–100%	10	10

Excluded	4	3

The gaps identified are surprisingly even more significant for those parameters where these hospitals represent the maximum quality level of trauma management in their countries.

The major problems regard first of all the registration and the complete storage of data concerning trauma care, in anyone of its phases. This audit is rarely applied in both centers, especially regarding to preclinical phase and transfers. Without accurate documentation of these periods, the respective trauma system can not be considered as homogenous, nor the process of care can be steady. Furthermore, we found severe lacks in hourly chart documentation in both centers, in recording the extubation/reintubation occurrence in the Bologna database and the complication rate in Bucharest database.

For the patient care, we noted inconsistencies with the current international standards, especially for surgical treatment of long bones fractures. The mortality rates are not comparable, since we could not consider the late mortality rate in the Italian group. However, the differences in the results between the two centers needs further studies.

The inappropriate transportation from the place of the crash and thus the lacking of prehospital care, the high number of head trauma in the Romanian group and the ten times higher number of transfers in the same group seemed to strongly influence the mortality rates.

In Romania, where nearly 70 percent of the patients are transferred in, many are delayed several to many hours after the injury. The outcome of these patients, who seem to have similar levels of injury as in the Italian patients, led to a doubling of mortality would argue that care delivery in the pre-hospital phase in Romania doubled mortality in these similarly injured patients. The most likely cause is a markedly different approach to care in the pre-hospital care in Italy versus Romania. But also the lacking of reliable data about pre-hospital phases prevent us from a precise analysis and definitive interpretation of the reported mortality data. The influence of pre-hospital care on survival in the two institutions should be tested in a further study.

Although our research proved good results respect to the ACS audits in both centers with relevant differences, mostly in the outcome, it showed also important gaps in the data registration and defects in early treatment of fractures. The presence of such defects in level I trauma centers is an early warning for all the health system and structures involved in trauma care in both countries. It is mandatory for these structures the improvement of the quality control, and it is the only way to establish the efficacy of the system.

Further investigation including prospective studies focusing on the improvement in the Trauma Care system as well as in the collection and interpretation of the data, are needed for achieving better outcomes in severely injured patients.

## Competing interests

The authors declare that they have no competing interests.

## Authors' contributions

SC, RS, GT and FB conceived of the study and participated in its design and coordination. RS, SC, GT and SDS carried out the study and drafted the manuscript. GT and SDS participated in the design of the study and performed the statistical analysis. GT, FB and SDS reviewed the manuscript. All authors read and approved the final manuscript
